# Going Viral: a Novel Role for Bacteriophage in Colorectal Cancer

**DOI:** 10.1128/mBio.02626-18

**Published:** 2019-01-22

**Authors:** Scott A. Handley, Suzanne Devkota

**Affiliations:** a Department of Medicine, Division of Gastroenterology, Cedars-Sinai Medical Center, Los Angeles, California, USA; b Department of Pathology and Immunology, Washington University School of Medicine, St. Louis, Missouri, USA

**Keywords:** bacteriophage, colorectal cancer, microbiome, virome

## Abstract

Microbiome-based signatures of disease have focused primarily on the bacterial component of the microbiome for numerous reasons, including ease of sample preparation and depth of the curated bacterial database. However, even more numerous than bacteria are the bacteriophages of the viral portion of the microbiome, which have emerged with identifiable disease signatures in other diseases, such as inflammatory bowel diseases. Here, G. D. Hannigan, M. B. Duhaime, M. T. Ruffin, IV, C. C. Koumpouras, and P. D. Schloss (mBio 9:e02248-18, https://doi.org/10.1128/mBio.02248-18) present a study that explores the potential bacteriophage signatures in patients with colorectal cancer (CRC) and the associated changes in bacterial signatures. Sampling from a cross section of 60 patients at different stages of CRC in addition to 30 healthy controls, this study highlights the need for greater exploration into the virome, including the “dark matter” of diverse forms that viruses assume in the gastrointestinal tract.

## Abstract (precis)

Microbiome-based signatures of disease have focused primarily on the bacterial component of the microbiome for numerous reasons, including ease of sample preparation and depth of the curated bacterial database. However, even more numerous than bacteria are the bacteriophages of the viral portion of the microbiome, which have emerged with identifiable disease signatures in other diseases, such as inflammatory bowel diseases.

## COMMENTARY

Survival rates after colorectal cancer (CRC) diagnosis have increased over the last decade due to multiple efforts by the media and physicians to encourage routine colonoscopies. In fact, a recent study looking at organized CRC screening, consisting of annual fecal immunochemical testing and colonoscopies, found a 52.4% reduction in cancer mortality (1). The authors found that this was due primarily to greater detection of early-stage cancers; therefore, proactive screening and early detection are key.

Detection of microbiome-based signatures of CRC have held promise as a complementary approach to colonoscopies because (i) shifts in gut bacterial community structure can often precede cellular changes in the host and (ii) the signatures of these shifts can be noninvasively measured in the stool. Detecting both of these would meaningfully aid in early CRC detection and would be especially helpful in at-risk populations.

The current study by Hannigan et al. (2) takes on this important challenge by asking whether the viral fraction of the microbiome (“virome”) may be an important influence in the human CRC microbiome and whether there are identifiable signatures between healthy individuals and those with precancerous and cancerous polyps. This study builds off the group’s earlier work (3), which focused on the bacterial component of the microbiome in the same human cohort. The microbiome’s influence on the etiopathogenesis of CRC has been additionally established by multiple lines of evidence, with consistently reported patterns, notably the presence of the bacterium Fusobacterium nucleatum in colon cancer patients and the ability of this bacterium to drive tumor progression in rodent models.

To achieve a cross-sectional picture of bacterial and viral communities across disease states, the authors collected stool samples from 30 patients with precancerous adenomas and 30 patients with diagnosed carcinoma, as well as 30 healthy control subjects. Standard exclusion criteria were applied, including no other preexisting comorbidities and treatment naiveté for CRC.

The interesting findings from this study are as much technical as they are biological. For example, stool as a representative of mucosal bacterial communities is often a controversial topic, but it is an accepted surrogate for assessing colonic microbial communities, and when attempting to develop noninvasive diagnostics, one must fully explore the opportunities that exist in noninvasive source material. Many clues in stool that have eluded investigators for myriad reasons may still exist. Indeed, in this study, the authors point out that standard alpha and beta diversity measurements failed to capture the virome diversity in the samples, while an operational taxonomic unit approach did. The observation that alpha and beta diversity measures may not be powerful enough to detect distinguishing features between states in microbiome data sets is not a new concept. In fact, research on the enteric virome and bacterial microbiome of lentivirus-infected humans and macaques led to similar conclusions; in those studies, diversity measures were incapable of resolving key features, while statistically rigorous tests were capable of associating a variety of enteropathogenic bacteria and viruses with lentivirus-induced immunodeficiency (4, 8). The Hannigan et al. study (2), while using a slightly different methodology, should encourage microbiome investigators to deeply interrogate their data beyond some of the more traditional measures used in microbial ecology in order to maximize their ability to detect significant associations.

Major biological findings from this study are both confirmatory and novel. Consistently with the bacterial literature, the authors found Fusobacterium nucleatum to be the bacterium most highly associated with tumor formation when they compared healthy patients versus adenoma patients and healthy patients versus carcinoma patients and then compared all three states. However, the dearth of virome data in CRC studies allows for novelty to be discovered around every corner and is why this study begins to fill a knowledge gap in the field. Of the identified viruses, the majority (78.8%) aligned with bacteriophage reference genomes and consisted primarily of *Siphoviridae*, *Myoviridae*, and several other unidentified bacteriophages. What is interesting when comparing the bacterial and viral signatures is that the CRC-associated bacterial signature tends to be dominated by a few key drivers, primarily *Fusobacterium*, while the CRC-associated virome signature appears to have greater diversity. Considering these things together, the authors propose an intriguing model wherein the dynamics between bacteriophages and bacteria are altered during a pathological state. This results in bacteriophage production, which alters bacterial communities in a predator-prey relationship, resulting in a biofilm scaffold made of dead microbial components. In turn, this opens up new niches for colonization by opportunistic bacteria, such as F. nucleatum, which may drive cancer progression. This disease-bacteriophage-bacterium-host dynamic has also been described in noncancerous inflammatory bowel disease (5), suggesting that transkingdom community dynamics should be considered part of the microbial circuitry underlying health and disease in the gut.

It should be noted that this study interrogated only a small portion of the virome. Viruses exist in several genetic forms that differ by the backbone nucleic acid (RNA or DNA), strandedness (positive or negative sense), and number of strands (single stranded versus double stranded). This complexity presents challenges in library preparation and sequencing strategy. The present study assessed only the double-stranded DNA (dsDNA) virome and therefore missed all RNA and single-stranded viruses. In addition, the authors use a protocol that biases toward enveloped viruses. Human enteric disease has a long history of interactions with viruses that would have been missed in this study (e.g., norovirus, rotavirus, astrovirus). The authors thoughtfully acknowledge several of these limitations and do not overstate their claims. These limitations do not diminish the findings of this study, and many other important studies have also decidedly focused on a single type of virus (4, 6). In addition, there is a growing appreciation for bacteriophages with RNA genomes (7). Thus, the findings reported here should be viewed as only the beginning of more comprehensive studies establishing the relationship between the enteric virome associated with CRC.

An underappreciated finding reported in this and other virome studies is our current deficiency in characterizing viral metagenomic data. Although many of the challenges in producing genetic material from RNA (i.e., cDNA synthesis) and single-stranded (i.e., second-strand synthesis) viruses can be met at the bench, the greater challenge resides in classifying viral metagenomic sequence data after it arrives from the sequencing facility. Hannigan et al. note that “as much as 95% of virus sequences belong to unknown genomic units” and that this is a common challenge in virome studies, with the amount of unclassifiable sequences (taxonomically or functionally) ranging from 70 to 90%. This unclassifiable fraction is frequently referred to as the “dark matter” of the virome. There are several reasons for our inability to accurately determine the provenance of sequences from viral preparations, and they include sparse reference databases and an incomplete understanding of how to assemble viral genomes from metagenomic data, as well as suboptimal algorithms for efficient classification. While these challenges will undoubtedly be met through additional efforts, they currently present the primary challenge to complete virome characterization. The authors’ findings are clear regarding what was classifiable, but it is intriguing to consider what might be hiding in the dark matter and how it may contribute to or be directly or indirectly associated with CRC and every other disease where the microbiome has been implicated.

The paper of Hannigan et al. offers a detailed and sophisticated exploration into the colorectal-cancer-associated virome and offers an intriguing theory on how bacteriophage-bacterium dynamics may promote a novel colonization niche for cancer-associated bacteria. It also transparently addresses the challenges and considerations of performing such studies. These findings open up an entirely new area of investigation into cancer biology as well as provide details on potential, noninvasive markers of cancer progression. While these findings are foundational for future research, much work still remains to be done by investigating virome-bacterial microbiome dynamics in larger, more disease stratified cohorts as well as on the complete characterization of the associated viromes.
